# Correlates of hospitalizations in internal medicine divisions among Israeli adults of different ethnic groups with hypertension, diabetes and cardiovascular diseases

**DOI:** 10.1371/journal.pone.0215639

**Published:** 2019-04-24

**Authors:** Shira Sagie, Wasef Na'amnih, Juda Frej, Daniel Cohen, Gershon Alpert, Khitam Muhsen

**Affiliations:** 1 Department of Epidemiology and Preventive Medicine, School of Public Health, Sackler Faculty of Medicine, Tel Aviv University, Ramat Aviv, Tel Aviv, Israel; 2 Clalit Health Services, Hadera, Israel; University of Malta Faculty of Health Sciences, MALTA

## Abstract

**Background:**

Disparities in non-communicable diseases (NCDs) may affect health care utilization. We compared the correlates of hospitalizations in internal medicine divisions, of adults with NCDs, between the main population groups in Israel.

**Methods:**

A cross-sectional study was conducted among Jews (N = 17,952) and Arabs (N = 10,441) aged ≥40 years with diabetes, hypertension or cardiovascular diseases, utilizing the computerized database of the largest health maintenance organization in Israel. Information was retrieved on sociodemographics, background diseases, hospitalizations and utilizations of other health services. Multivariable log binomial regression models were performed.

**Results:**

Overall, 3516 (12.4%) patients were hospitalized at least once during a one-year period (2008). Hospitalization in internal medicine divisions was more common among Arab than Jewish patients; prevalence ratio 1.24 (95% CI 1.14–1.35), and increased with age (P<0.001). An inverse association was found between residential socioeconomic status and hospitalization among Jewish patients, but not among Arab, who lived mostly in low socioeconomic status communities. In both population groups, congestive heart failure, arrhythmias, heart surgery, cardiac catheterization, kidney disease, asthma, neurodegenerative diseases, mental illnesses, smoking (in men) and disability were positively related to hospitalization in internal medicine divisions, which was more common also in patients who consulted any specialist and a specialist in cardiology. Emergency room visits, consulting with an ophthalmologist and performing cancer screening tests were inversely related to hospitalizations among Jewish patients only (P = 0.009 and P = 0.067 for interaction, respectively).

**Conclusions:**

In a country with universal health insurance, the correlates of hospitalizations included sociodemographics, multi-morbidity, health behaviors and health services use patterns. Socioeconomic disparities might account for ethnic differences in hospitalizations. Individuals with several NCDs, rather than one specific disease, disability and smoking should be targeted to reduce healthcare costs related to hospitalizations.

## Introduction

Non-communicable diseases (NCDs), including cardiovascular diseases (CVD), cancer, chronic respiratory diseases and diabetes, account for 70% of deaths worldwide; CVD is considered the cause of 31% of all deaths [1]. Behavioral risk factors such as physical inactivity, smoking and poor diet explain nearly 80% of the CVD burden, making these diseases and their risk factors a main target for interventions [2]. Disparities in health and in NCDs exist between and within countries, and they are affected by social determinants, including education, income and ethnicity [3–8].

In Israel, cancer, heart disease, stroke and diabetes are currently the leading causes of death; nearly 50% of all deaths in 2016 were attributed to these conditions [9]. We and others have demonstrated health disparities between the two main population groups in Israel, the Jewish and Arab populations, in NCD morbidity and its related risk factors and mortality [10–12]. Despite the continuous increase in both populations, life expectancy remains consistently lower among the Arab compared to the Jewish population, and the gap in life expectancy has been widening over recent years [10]. In 2017, life expectancy at birth was 3.7 years lower in Arab than Jewish men: 77.5 and 81.2 years, respectively, and 3.0 years lower in Arab vs. Jewish women; 82.0 vs. 85.0 years [13]. The higher mortality rates of heart diseases, stroke and diabetes among the Arab population [10] contribute substantially to the gap in life expectancy between the two population groups [14, 15].

Disparities between Arabs and Jews in Israel were also shown in health care utilization [12, 16, 17], despite the universal health insurance law and high access to care in both populations. Findings from national health surveys have shown higher age-adjusted rates of hospitalizations among Arabs than Jews. Such differences were observed in nearly all age-strata for both sexes [16, 17]. Interestingly, Arab participants reported visiting a family physician more often than Jewish participants, while the latter more often visited specialist physicians [16–18]. Clearly, utilization of health services is affected by health status; however, additional factors were also found to be important, including sex, age, income, social/health insurance status and language barriers [19–21]. We showed a significantly higher age-adjusted rate of hospitalization in internal medicine divisions among Arabs with NCDs than among Jews living in the same region [22]. The aim of the current study was to compare the correlates of hospitalizations in internal medicine divisions between Arab and Jewish patients with chronic diseases: CVD, diabetes and hypertension, in Israel. Our hypothesis was that demographic, clinical and behavioral characteristics (e.g. comorbidities, health care utilization patterns) are related to hospitalizations, and that these might differ according to ethnic group.

## Materials and methods

### Study design and population

A cross-sectional study was conducted of all adults aged 40 years or above from the Hadera sub-district, utilizing the database of the Sharon-Shomron sub-district of Clalit Health Services [23]. Clalit is the largest health maintenance organization (HMO) in Israel, insuring about 4.2 million members; i.e. ~52% of the Israeli population. The Hadera sub-district comprises 51.8% and 45.7% Jews and Arabs, respectively, compared with 75.5% and 20.4%, respectively, in the general population in Israel. Study eligibility criteria included: having CVD, hypertension or diabetes, as documented in the Clalit database by primary care physicians’ reports at any time before or during 2008 (S1 and S2 Tables) [24]. This yielded a total of 31,883 persons. We excluded from the study 3471 patients with a diagnosis of cancer and 19 patients without information on ethnicity.

### Definitions of the dependent variable

The dependent variable was defined as at least one hospitalization in an internal medicine division during the study period year (2008) (yes or no), based on the documentation in the Clalit database. Hospitalizations in internal medicine divisions included: internal medicine departments, intensive care units, cardiac intensive care units and neurology departments. Documentation of hospitalizations is based on payments of Clalit Health Services to the hospitals for these services, therefore the accuracy is high.

### Definitions of the independent variables (S1 Table)

#### Sociodemographic variables

The main independent variable was population (ethnic) group, Jews or Arabs, which was defined based on place of residence: Arab or Jewish town/city. Additional variables were sex and age (as a continuous variable in years, and as a categorical variable grouped as 40–44, 45–49, 50–54, 55–59, 60–64, 65–69, 70–74 and ≥75 years). Socioeconomic status (SES) was defined using the socioeconomic rank of place of residence according to the classification of the Central Bureau of Statistics. The ranks are on a scale from 1 to 10, with higher ranks representing a higher SES. This aggregative socioeconomic index reflects a combination of financial characteristics of a specific geographical unit, e.g. housing conditions, motorization, education and employment [25].

#### Health related variables

Information was collected on background diseases, physical disability, smoking and obesity, based on diagnoses recorded in medical records. Ischemic heart disease (IHD) was defined in the Clalit database. CVD was defined as having any of the following diagnoses: IHD, congestive heart failure (CHF), arrhythmia, pulmonary hypertension, cardiomyopathy, carotid artery disease or stroke. A comorbidity summative score was calculated (S2 Table). We obtained information about the number of cardiac interventions (heart surgeries and cardiac catheterizations). We obtained data on health care utilization including performing cancer screening tests (stool occult blood, prostatic specific antigen [PSA] for men and mammography for women), influenza vaccination and visits to specialist physicians.

### Data analysis

The study sample was described using descriptive statistics. A bivariate analysis comparing hospitalizations in internal medicine divisions according to the independent variables (sociodemographics and background morbidity) was performed using chi square test and Fisher Exact test as appropriate, for categorical variables, the Student’s *t-*test for continuous variables, and the Mann-Whitney test for variables that did not follow normal distributions. For each independent variable, prevalence ratios (PR) and 95% confidence intervals (CI) were obtained from generalized linear models with log binomial regression models. Pooled and population stratified analyses were performed. Heterogeneity of the PRs according to population group was assessed using the chi square test for heterogeneity. Multivariable analyses were performed using generalized linear models with log binomial regression models, from which adjusted PR (and 95% CI) were obtained for each independent variable. Interactions between each independent variable and population group were assessed in multivariable models. The Akaike information criterion was used to compare models. Correlations between independent variables were assessed by Spearman's correlation coefficient for ordinal variables and Phi correlation coefficient for nominal variables. Independent variables with correlation coefficients of 0.6 or greater were examined in separate models. Collinearity between independent variables was assessed using the variance inflation factor (VIF). P<0.05 was considered statistically significant. Data were analyzed using SPSS version 25 (IBM, New York, United States)

### Ethics

The study was approved by the ethical review committee of Clalit Health Services. The data used in the current study were anonymized.

## Results

During the study period (2008), there were 28,393 patients (mean age 63.1 years standard deviation [SD] 12.3), 52.2% women) who met the study inclusion criteria. The study sample consisted of 63.2% Jewish patients and 36.8% Arab patients. The mean age of the Arab patients was lower than that of Jewish patients by 7 years: 58.3 (SD 11.3) and 65.9 (SD 12.1) years, respectively (P<0.001). Arab and Jewish patients were comparable regarding sex distribution (53.8% and 51.3% were women, respectively). Arab patients lived in towns of lower SES rank than Jewish participants (mean 3.0 [SD 0.9] vs. 6.0 [SD 0.5], P<0.001).

Overall, hypertension, IHD and diabetes were documented in 74.8%, 26.9% and 48.6% of the study sample. The respective percentages among Jewish patients were 78.4%, 27.5% and 42.5%; and among Arab patients 68.5%, 25.9% and 58.9% (P<0.001 for all comparisons between Arab and Jewish patients).

A total of 3516 (12.4%) patients had at least one hospitalization in an internal medicine division during the study period, 11.7% and 13.6% among Jewish and Arab patients, respectively (P<0.001). In the Jewish sample this percentage was the same (11.7%) among men and women, compared to 15.2% and 12.1% among Arab men and women, respectively (P<0.001 for the difference between Arab men and women) (P<0.001 for heterogeneity).

### Correlates of hospitalizations in an internal medicine division (pooled analysis)

In a pooled analysis, hospitalization in internal medicine divisions increased with age (P for trend <0.001) and was 13.0% in men vs. 11.8% in women (Table 1). The mean rank of residential SES was lower in the hospitalized than the non-hospitalized group: 4.6 (SD 1.6) vs. 4.8 (1.6) (P<0.001).

**Table 1:** Hospitalizations in internal medicine divisions according to demographics, background diseases and utilization of other health care services ^a^.

			Overall			Jews			Arabs	
Demographic variables		Total	Hospitalized n (%)	p	Total	Hospitalized n (%)	p	Total	Hospitalized n (%)	p
**Sex**	Men	13,576	1761 (13.0)		8750	1025 (11.7)		4826	736 (15.3)	
	Women	14,817	1755 (11.8)	0.01	9202	1075 (11.7)	0.9	5615	680 (12.1)	<0.001
**Age, years**				<0.001			<0.001			<0.001
	40–44	1665	134 (8.0)		517	40 (7.7)		1148	94 (8.2)	
	45–49	2493	195 (7.8)	0.8	975	68 (7.0)	0.6	1518	127 (8.4)	0.9
	50–54	3501	322 (9.2)	0.2	1794	141 (7.9)	0.9	1707	181 (10.6)	0.051
	55–59	4323	384 (8.9)	0.4	2657	213 (8.0)	0.8	1666	171 (10.3)	0.09
	60–64	4201	468 (11.1)	0.001	2892	267 (9.2)	0.3	1309	201 (15.4)	<0.001
	65–69	3391	420 (12.4)	<0.001	2122	195 (9.2)	0.3	1269	225 (17.7)	<0.001
	70–74	3163	474 (15.0)	<0.001	2307	285 (12.4)	0.008	856	189 (22.1)	<0.001
	≥75	5656	1119 (19.8)	<0.001	4688	891 (19.0)	<0.001	968	228 (23.6)	<0.001
**Morbidity**										
Hypertension	No	7160	690 (9.6)		3872	368 (9.5)		3288	322 (9.8)	
	Yes	21,233	2826 (13.3)	<0.001	14,080	1732 (12.3)	<0.001	7153	1094 (15.3)	<0.001
Diabetes	No	14,603	1647 (11.3)		10,316	1121 (10.9)		4287	526 (12.3)	
	Yes	13,970	1869 (13.6)	<0.001	7636	979 (12.8)	<0.001	6157	890 (14.5)	0.005
IHD	No	20,742	1780 (8.6)		13,009	1107 (8.5)		7733	673 (8.7)	
	Yes	7651	1736 (22.7)	<0.001	4943	993 (20.1)	<0.001	2708	743 (27.4)	<0.001
CHF	No	26,986	2907 (10.8)		17,195	1790 (10.4)		9791	1117 (11.4)	
	Yes	1407	609 (43.3)	<0.001	757	310 (41.0)	<0.001	650	299 (46.0)	<0.001
Cardiomyopathy	No	27,991	3393 (12.1)		17,716	2033 (11.5)		10,275	1360 (13.2)	
	Yes	402	123 (30.6)	<0.001	236	67 (28.4)	<0.001	166	56 (33.7)	<0.001
Arrhythmia	No	25,428	2572 (10.1)		15,936	1488 (9.3)		9492	1084 (11.4)	
	Yes	2965	944 (31.8)	<0.001	2016	612 (30.4)	<0.001	949	332 (35.0)	<0.001
Carotid artery disease	No	27,448	3301 (12.0)		17,147	1942 (11.3)		10,301	1359 (13.2)	
	Yes	945	215 (22.8)	<0.001	805	158 (19.6)	<0.001	140	59 (40.7)	<0.001
Stroke	No	25,644	2706 (10.6)		16,145	1618 (10.0)		9499	1088 (11.5)	
	Yes	2749	810 (29.5)	<0.001	1807	482 (26.7)	<0.001	942	328 (34.8)	<0.001
CVD	No	17,589	1032 (5.9)		10,870	635 (5.8)		6719	397 (5.9)	
	Yes	10,804	2484 (23.0)	<0.001	7082	1465 (20.7)	<0.001	3722	1019 (27.4)	<0.001
Cardiac catheterization	No	26,048	2719 (10.4)		16,552	1693 (10.2)		9496	1026 (10.8)	
	Yes	2345	797 (34.0)	<0.001	1400	407 (29.1)	<0.001	945	390 (41.3)	<0.001
Past heart surgery	No	28,243	3428 (12.1)		17,861	2051 (11.5)		10,382	1377 (13.3)	
	Yes	150	88 (58.7)	<0.001	91	49 (53.8)	<0.001	59	39 (66.1)	<0.001
Kidney disease	No	26,893	2976 (11.1)		16,897	1777 (10.5)		9996	1199 (12.0)	
	Yes	1500	540 (36.0)	<0.001	1055	323 (30.6)	<0.001	445	217 (48.8)	<0.001
Mental illness	No	24,477	2736 (11.2)		14,803	1505 (10.2)		9674	1231 (12.7)	
	Yes	3916	780 (19.9)	<0.001	3149	595 (18.9)	<0.001	767	185 (24.1)	<0.001
Neurodegenerative disease	No	27,358	3217 (11.8)		17,103	1862 (10.9)		10255	1355 (13.2)	
	Yes	1035	299 (28.9)	<0.001	849	238 (28.0)	<0.001	186	61 (32.8)	<0.001
Hyperlipidemia	No	7047	633 (9.0)		4272	393 (9.2)		2775	240 (8.6)	
	Yes	21,346	2883 (13.5)	<0.001	13,680	1707 (12.5)	<0.001	7666	1176 (15.3)	<0.001
Asthma	No	26,073	3097 (11.9)		16,482	1859 (11.3)		9591	1238 (12.9)	
	Yes	2320	419 (18.1)	<0.001	1470	241 (16.4)	<0.001	850	178 (20.9)	<0.001
Disability	No	26,503	2879 (10.9)		16,786	1726 (10.3)		9717	1153 (11.9)	
	Yes	1890	637 (33.7)	<0.001	1166	374 (32.1)	<0.001	724	263 (36.3)	<0.001
**Health behavior and health care utilization patterns**										
Obesity	No	17,665	1996 (11.3)		12,392	1360 (11.0)		5273	636 (12.1)	
	Yes	10,728	1520 (14.2)	<0.001	5560	740 (13.3)	<0.001	5168	780 (15.1)	<0.001
Smoking	No	19,484	2138 (11.0)		12,747	1397 (11.0)		6737	741 (11.0)	
	Yes	8909	1378 (15.5)	<0.001	5205	703 (13.5)	<0.001	3704	675 (18.2)	<0.001
Received influenza vaccine	No	15,387	1412 (9.2)		9930	934 (9.4)		5457	478 (8.8)	
	Yes	13,006	2104 (16.2)	<0.001	8022	1166 (14.5)	<0.001	4984	938 (18.8)	<0.001
Performed HbA1c test	No	2182	222 (10.2)		1273	132 (10.4)		909	90 (9.9)	
	Yes	14,616	2066 (14.1)	<0.001	8407	1129 (13.4)	<0.001	6209	937 (15.1)	<0.001
Consulted a specialist	No	6549	491 (7.5)		3800	272 (7.2)		2749	219 (8.0)	
	Yes	21,844	3025 (13.8)	<0.001	14,152	1828 (12.9)	<0.001	7692	1197 (15.6)	<0.001
Consulted a diabetes specialist	No	26,098	3089 (11.8)		16,717	1908 (11.4)		9381	1181 (12.6)	
	Yes	2295	427 (18.6)	<0.001	1235	192 (15.5)	<0.001	1060	235 (22.2)	<0.001
Consulted a cardiologist	No	22,072	1870 (8.5)		13,636	1122 (8.2)		8436	748 (8.9)	
	Yes	6321	1646 (26.0)	<0.001	4316	978 (22.7)	<0.001	2005	668 (33.3)	<0.001
Consulted an ophthalmologist	No	16,883	1955 (11.6)		10,482	1183 (11.3)		6401	772 (12.1)	
	Yes	11,510	1561 (13.6)	<0.001	7470	917 (12.3)	0.071	4040	644 (15.9)	<0.001
Consulted a surgeon	No	20,995	2429 (11.6)		12,564	1354 (10.8)		8431	1075 (12.8)	
	Yes	7398	1087 (14.7)	<0.001	5388	746 (13.8)	<0.001	2010	341 (17.0)	<0.001
Performed a cancer screening test	No	16,657	2146 (12.9)		9696	1236 (12.7)		6961	910 (13.1)	
	Yes	11,736	1370 (11.7)	0.007	8256	864 (10.5)	<0.001	3480	506 (14.5)	0.07
Emergency department visit	No	23,459	2365 (10.1)		15,154	1467 (9.7)		8305	898 (10.8)	
	Yes	4934	1151 (23.3)	<0.001	2798	633 (22.6)	<0.001	2136	518 (24.3)	<0.001

^a^ The data presented are absolute numbers and percentages in parentheses. P values were obtained by the chi square test.

CHF: congestive heart failure; CVD: cardiovascular disease; IHD: ischemic heart disease.

Hospitalizations in internal medicine divisions were significantly more common in patients who had any of the examined background diseases (P<0.001) than in those who did not (Table 1). The magnitude of the associations differed for the various conditions, with unadjusted PR ranging between 1.20 (95% CI 1.12–1.29) for patients who had diabetes compared to those who did not, to a PR of 4.03 (95% CI 3.47–4.69) for patients who had CHF compared to those who did not (Table 2). The hospitalized group had a significantly higher mean comorbidity score (4.8 [SD 1.9] than the non-hospitalized group (3.4 [SD 1.5]) P<0.001. Hospitalizations in internal medicine divisions were significantly more common in smokers and patients with obesity than in those without such conditions. Hospitalization in internal medicine divisions was more common in patients who utilized other health care services (except for cancer screening tests) than those who did not. Among patients who consulted with a cardiologist hospitalization in an internal medicine division was 26.0% among patients who consulted with a cardiologist vs. 8.5% among patients who did not consult (Table 1).

**Table 2:** Unadjusted associations of sociodemographic variables, background diseases and health care utilization pattern with hospitalizations in internal medicine divisions ^a^.

	Overall	Jews	Arabs	
	PR (95% CI)	PR (95% CI)	PR (95% CI)	P for heterogeneity
**Sociodemographic variables**				
**Sex**, Women vs. men	0.91 (0.85–0.98)	1.00 (0.91–1.09)	0.79 (0.71–0.89)	0.001
**Age, years (reference age 40–44)**				<0.001
45–49	0.97 (0.77–1.22)	0.90 (0.60–1.35)	1.02 (0.77–1.35)	0.6
50–54	1.14 (0.93–1.41)	1.02 (0.71–1.46)	1.30 (0.99–1.68)	0.3
55–59	1.10 (0.90–1.35)	1.04 (0.73–1.48)	1.25 (0.96–1.63)	0.4
60–64	1.38 (1.13–1.69)	1.19 (0.85–1.69)	1.88 (1.45–2.43)	0.04
65–69	1.54 (1.26–1.89)	1.19 (0.83–1.69)	2.17 (1.68–2.79)	0.007
70–74	1.86 (1.52–2.28)	1.60 (1.13–2.25)	2.70 (2.07–3.51)	0.02
≥75	2.46 (2.04–2.97)	2.46 (1.76–3.42)	2.88 (2.23–3.71)	0.5
Residential SES, mean (SD)	0.96 (0.94–0.98)	0.85 (0.78–0.93)	0.98 (0.92–1.04)	0.009
**Background morbidity**				
Hypertension	1.38 (1.27–1.51)	1.29 (1.15–1.46)	1.56 (1.37–1.78)	<0.001
IHD	1.20 (1.12–1.29)	1.18 (1.04–1.29)	1.18 (1.05–1.32)	0.4
Diabetes	2.64 (2.46–2.84)	2.36 (2.15–2.59)	3.15 (2.82–3.53)	<0.001
CHF	4.03 (3.47–4.69)	3.93 (3.42–4.53)	4.03 (3.47–4.69)	0.8
Cardiomyopathy	2.55 (1.87–3.47)	2.47 (1.88–3.26)	2.55 (1.87–3.47)	0.9
Arrhythmia	3.06 (2.66–3.52)	3.25 (2.93–3.61)	3.06 (2.66–3.52)	0.5
Carotid artery disease	3.09 (2.26–4.22)	1.73 (1.45–2.07)	3.09 (2.26–4.22)	0.002
Stroke	2.79 (2.56–3.05)	2.66 (2.38–2.98)	3.04 (2.64–3.50)	0.006
CVD	3.35 (3.12–3.61)	3.05 (2.78–3.35)	3.93 (3.49–4.41)	<0.001
Cardiac catheterization	3.26 (2.98–3.56)	2.84 (2.52–3.21)	3.82 (3.34–4.37)	<0.001
Past heart surgery	4.83 (3.71–6.30)	4.69 (3.30–6.66)	4.98 (3.31–7.50)	0.3
Kidney disease	3.25 (2.93–3.62)	2.91 (2.55–3.33)	4.07 (3.42–4.83)	<0.001
Mental illness	1.78 (1.63–1.94)	1.86 (1.69–2.06)	1.90 (1.60–2.25)	0.1
Neurodegenerative disease	2.46 (2.15–2.81)	2.58 (2.21–3.00)	2.48 (1.85–3.33)	0.7
Hyperlipidemia	1.51 (1.37–1.65)	1.36 (1.21–1.52)	1.77 (1.53–2.05)	<0.001
Asthma	1.52 (1.36–1.70)	1.45 (1.26–1.68)	1.62 (1.37–1.93)	0.2
Disability	3.10 (2.81–3.42)	3.12 (2.75–3.54)	3.06 (2.63–3.57)	0.8
Comorbidity score, median (IQR)	1.44 (1.41–1.47)	1.41 (1.37–1.44)	1.49 (1.44–1.53)	<0.001
**Health behavior and health care utilization patterns**				
Obesity	1.25 (1.17–1.35)	1.21 (1.10–1.33)	1.25 (1.12–1.40)	0.6
Smoking	1.41 (1.31–1.52)	1.23 (1.12–1.36)	1.66 (1.48–1.85)	<0.001
Received influenza vaccine	1.76 (1.64–1.89)	1.55 (1.49–1.69)	2.15 (1.91–2.41)	<0.001
Performed HbA1c test	1.39 (1.20–1.61)	1.30 (1.07–1.57)	1.52 (1.21–1.91)	0.3
Consulted a specialist	1.85 (1.67–2.04)	1.81 (1.58–2.06)	1.95 (1.68–2.27)	0.4
Consulted a diabetes specialist	1.57 (1.41–1.75)	1.36 (1.16–1.60)	1.76 (1.51–2.05)	0.008
Consulted a cardiologist	3.07 (2.86–3.30)	2.75 (2.51–3.02)	3.76 (3.35–4.22)	<0.001
Consulted an ophthalmologist	1.17 (1.09–1.26)	1.09 (0.99–1.19)	1.32 (1.18–1.48)	0.001
Consulted a surgeon	1.27 (1.18–1.37)	1.29 (1.17–1.41)	1.33 (1.17–1.52)	0.3
Performed a cancer screening test	0.91 (0.84–0.97)	0.82 (0.75–0.90)	1.11 (0.99–1.25)	<0.001
Emergency department visit	2.31 (2.14–2.50)	2.34 (2.11–2.59)	2.24 (1.99–2.52)	0.6

P for heterogeneity by population group was calculated by the chi square test for heterogeneity.

^a^ CHF: congestive heart failure; CI: confidence intervals; CVD: cardiovascular disease; IHD: ischemic heart disease; IQR: interquartile range; PR: prevalence ratio; SD: standard deviation.

### Correlates of hospitalizations in internal medicine divisions by population group

In the Jewish sample, the percentage of patients who were hospitalized in internal medicine divisions was relatively low (7.0%-9.2%) at ages 40–69 years, but increased to 12.4% in patients aged 70–74 years and 19.0% in those aged ≥75 years. In the Arab sample, percentages of hospitalized patients were 8.2%-10.3% at ages 40–59 years and increased substantially to 15.4% and 23.6% in the older age groups (P<0.001 for heterogeneity) (Tables 1 and 2). Among Jewish patients, residential SES was inversely related to hospitalizations, but not among Arabs (Table 2).

In both the Arab and Jewish samples, hospitalizations in internal medicine divisions were more common among patients who had any of the examined background diseases, obesity, smoking or disability than among patients who did not (Table 1). However, the magnitude of the association was stronger among Arabs for CVD, kidney disease hyperlipidemia and smoking (P<0.05 for heterogeneity) (Table 2). The hospitalized group in the Jewish sample had a significantly higher mean comorbidity score (4.6 [SD 1.9] than the non-hospitalized group (3.4 [SD 1.5]) P<0.001. The respective values among Arab patients were 4.9 (SD 1.9) and 3.4 (SD 1.5), P<0.001.

In both population groups, significant positive associations were found between influenza vaccination, consulting a specialist and visiting the emergency room department, with hospitalizations in internal medicine divisions (Table 1), but the associations with influenza vaccine and consulting a diabetes specialist, cardiologist and ophthalmologist were of stronger magnitude among Arabs (P<0.05 for heterogeneity). Significant heterogeneity was also found in relation to performing cancer-screening tests, which was inversely related to hospitalizations in internal medicine divisions among Jewish patients only (Table 2).

### Multivariable analysis of the correlates of hospitalizations in internal medicine divisions

The variables, residential SES and population group, were highly correlated (Spearman's coefficient 0.89); therefore, they were not included in the same model, and separate analyses were performed for each population group. Weak correlations were found between some other independent variables (S1 Fig).

In an initial pooled multivariable analysis that included both population groups, hospitalization in internal medicine divisions was more common among Arab than Jewish patients: adjusted PR 1.24 (95% CI 1.14–1.35). Age was positively associated with hospitalizations in internal medicine divisions: adjusted PR 1.02 (95% CI 1.01–1.03) for each 1-year increase, but no significant difference was found between men and women (P = 0.19). Hospitalization in internal medicine divisions was significantly higher, by 1.52 to 1.75-fold, in patients who had CHF, arrhythmia, stroke, cardiac catheterization, past heart surgery or disability than in patients who did not have such conditions. Associations of having kidney disease, asthma, neurodegenerative diseases and mental illnesses with hospitalization in internal medicine divisions were also significant, although these were of smaller magnitude, with adjusted PR of 1.28–1.46. Hospitalization in internal medicine divisions was 1.91-fold more common (P<0.001) in patients who consulted with a cardiologist and 1.67-fold higher (P<0.001) in patients who had visited the emergency room department than in patients who did not. Consulting any specialist or a diabetes specialist, and also smoking and obesity, demonstrated positive significant associations (adjusted PR of 1.11 to 1.30) with hospitalization in internal medicine divisions; while inverse associations were found with consulting an ophthalmologist (adjusted PR 0.89 [95% CI 0.82–0.97]) and performing a cancer screening test (adjusted PR 0.85 [95% CI 0.79–0.93]) (Table 3).

**Table 3:** Multivariable analysis of the correlates of hospitalizations in internal medicine divisions among patients aged ≥40 years with cardiovascular disease, diabetes or hypertension ^a^.

	Adjusted PR (95% CI)	P
**Sociodemographic variables**		
Population group (Arab vs. Jewish patients)	1.24 (1.14–1.35)	<0.001
Sex (women vs. men)	1.06 (0.97–1.16)	0.19
Age in years (a continuous variable)	1.02 (1.01–1.03)	<0.001
**Background morbidity**		
Diabetes	1.08 (0.99–1.17)	0.06
IHD	1.10 (1.01–1.21)	0.048
CHF	1.52 (1.35–1.72)	<0.001
Arrhythmia	1.60 (1.45–1.76)	<0.001
Stroke	1.61 (1.45–1.78)	<0.001
Cardiac catheterization	1.75 (1.57–1.96)	<0.001
Past heart surgery	1.52 (1.14–2.04)	0.004
Kidney disease	1.46 (1.29–1.65)	<0.001
Neurodegenerative disease	1.44 (1.23–1.68)	<0.001
Asthma	1.28 (1.13–1.44)	<0.001
Mental illness	1.36 (1.24–1.50)	<0.001
Disability	1.62 (1.43–1.83)	<0.001
**Health behaviors and health care utilization**		
Obesity	1.16 (1.07–1.25)	<0.001
Smoking	1.30 (1.19–1.42)	<0.001
Influenza vaccination	1.11 (1.02–1.20)	0.02
Consulted a specialist	1.30 (1.16–1.47)	<0.001
Consulted a diabetes specialist	1.16 (1.02–1.32)	0.02
Consulted a cardiologist	1.91 (1.75–2.09)	<0.001
Consulted an ophthalmologist	0.89 (0.82–0.97)	0.008
Performed a cancer screening test	0.85 (0.79–0.93)	<0.001
Emergency department visit	1.67 (1.54–1.82)	<0.001

^a^ CI: confidence intervals; CHF: congestive heart failure; IHD: ischemic heart disease; PR: prevalence ratio

Adjusted for the variables in the table.

In a model that included residential SES as independent variable instead of population group, no significant association was found between residential SES and hospitalizations in internal medicine divisions: adjusted PR 0.89 (95% CI 0.77–1.02), P = 0.08. Associations of the other independent variables with hospitalizations in internal medicine departments were similar to those obtained in the initial model (S3 Table). In both models, the VIF values ranged between 1.02 and 1.49, suggesting no collinearity between the independent variables.

Additional multivariable models that included the same independent variables as in Table 2, and also an interaction term between the variable "population group" and one independent variable, showed significant interactions with IHD, influenza vaccination, consulting with an ophthalmologist and performing a cancer screening test. No significant (P = 0.7) association was observed between having a diagnosis code of IHD and hospitalization in an internal medicine division among Jewish patients; however, Arab patients with a diagnosis code of IHD had 1.23-fold (95% CI 1.05–1.43) increased likelihood of hospitalization than Arabs lacking such a diagnosis code or than Jewish patients (P = 0.009 for interaction). Having received an influenza vaccination was not associated with hospitalization in internal medicine divisions in Jewish patients (P = 0.9); however, Arab patients who were vaccinated were hospitalized more often than those who were not vaccinated or than Jewish patients (adjusted PR 1.28 95% CI 1.09–1.50) (P = 0.002 for interaction). Having consulted with an ophthalmologist was inversely related to hospitalizations among Jewish patients (adjusted PR 0.83 [95% CI 0.75–0.91]), but not among Arab patients (adjusted PR 1.23 [95% CI 1.05–1.43]), (P = 0.009 for interaction). Similarly, performing a cancer screening test was inversely related to hospitalizations in internal medicine divisions among Jewish patients (adjusted PR 0.81 [95% CI 0.73–0.89]), but not among Arab patients (adjusted PR 1.16 [95% CI 0.99–1.36]), (P = 0.067 for interaction) (Table 4 and S4 Table). No significant interactions were found of population group or patient sex with other independent variables. Multivariable models conducted separately for each population group and for each sex showed similar findings (Table 5).

**Table 4:** Pooled multivariable models of the correlates of hospitalizations in internal medicine divisions with interaction terms ^a^.

	Adjusted PR (95% CI)	P
**Analysis 1**		
Population group, Arab vs. Jewish patients (without a diagnosis code of IHD)	1.13 (1.02–1.26)	0.025
IHD in Jewish patients	1.02 (0.91–1.14)	0.7
Interaction population group 1 = Arabs ˟IHD	1.23 (1.05–1.43)	0.009
**Analysis 2**		
Population group, Arab vs. Jewish patients with no influenza vaccination	1.08 (0.95–1.22)	0.2
Influenza vaccination in Jewish patients	1.00 (0.90–1.11)	0.9
Interaction population group 1 = Arabs˟ influenza vaccination	1.28 (1.09–1.50)	0.002
**Analysis 3**		
Population group, Arab vs. Jewish patients who did not consult with an ophthalmologist	1.13 (1.02–1.27)	0.02
Consulted an ophthalmologist, Jewish patients	0.83 (0.75–0.91)	<0.001
Interaction population group 1 = Arabs˟ consulted an ophthalmologist	1.23 (1.05–1.43)	0.009
**Analysis 4**		
Population group, Arab vs. Jewish patients who did not perform a cancer screening test	1.17 (1.05–1.30)	0.004
Performed a cancer screening test, Jewish patients	0.81 (0.73–0.89)	<0.001
Interaction population group 1 = Arabs˟ performed a cancer screening test	1.16 (0.99–1.36)	0.067

^a^ CI: confidence intervals; IHD: ischemic heart disease; PR: prevalence ratio.

Each model included the following variables: age, sex, population group, IHD, congestive heart failure, arrhythmia, stroke, cardiac catheterization, past heart surgery, kidney disease, neurodegenerative disease, asthma, mental illness, disability, obesity, smoking, influenza vaccination, consulting a specialist, consulting a diabetes specialist, consulting a cardiologist, consulting with an ophthalmologist, performing a cancer screening test, emergency department visit, and the interaction terms presented in the table. The full models are presented in S4 Table.

**Table 5:** Multivariable analysis of the correlates of hospitalizations in internal medicine divisions by population and sex groups ^a^.

	Jewish men		Arab men		Jewish women		Arab women	
	Adjusted PR (95% CI)	P	Adjusted PR (95% CI)	P	Adjusted PR (95% CI)	P	Adjusted PR (95% CI)	P
**Sociodemographic variables**								
SES place of residence	0.89 (0.77–1.02)	0.08	1.00 (0.91–1.10)	0.9	0.85 (0.75–0.97)	0.02	1.01 (0.91–1.11)	0.8
Age in years (a continuous variable)	1.01 (1.01–1.02)	0.03	1.01 (0.99–1.02)	0.16	1.02 (1.01–1.03)	<0.001	1.02 (1.01–1.03)	<0.001
**Background morbidity**								
Diabetes	1.12 (0.95–1.31)	0.17	0.95 (0.79–1.15)	0.6	1.10 (0.95–1.29)	0.2	1.17 (0.95–1.42)	0.14
IHD	1.06 (0.89–1.27)	0.5	1.18 (0.95–1.46)	0.13	1.16 (0.98–1.39)	0.09	1.13 (0.91–1.42)	0.2
CHF	1.55 (1.22–1.97)	<0.001	1.31 (1.02–1.68)	0.04	1.56 (1.21–2.02)	0.001	1.53 (1.16–2.02)	0.003
Arrhythmia	1.59 (1.31–1.92)	<0.001	1.53 (1.21–1.92)	<0.001	1.63 (1.36–1.95)	<0.001	1.53 (1.21–1.94)	<0.001
Stroke	1.61 (1.33–1.95)	<0.001	1.57 (1.23–2.02)	<0.001	1.51 (1.25–1.83)	<0.001	1.96 (1.55–2.48)	<0.001
Cardiac catheterization	1.49 (1.21–1.82)	<0.001	2.04 (1.65–2.53)	<0.001	1.59 (1.21–2.09)	0.001	1.89 (1.40–2.55)	0.001
Past heart surgery	1.78 (1.11–2.83)	0.02	1.53 (0.91–2.56)	0.11	1.31 (0.57–3.01)	0.5	0.89 (0.39–2.10)	0.7
Kidney disease	1.47 (1.19–1.82)	<0.001	1.58 (1.19–2.09)	0.002	1.47 (1.15–1.89)	0.002	1.62 (1.18–2.21)	0.003
Neurodegenerative disease	1.53 (1.13–2.06)	0.005	1.69 (1.05–2.73)	0.03	1.32 (1.03–1.69)	0.03	1.47 (0.92–2.33)	0.11
Asthma	1.33 (1.02–1.73)	0.03	1.32 (0.99–1.75)	0.057	1.43 (1.17–1.76)	0.001	1.21 (0.93–1.56)	0.16
Mental illness	1.28 (1.06–1.54)	0.009	1.39 (1.05–1.84)	0.02	1.40 (1.20–1.63)	<0.001	1.42 (1.09–1.85)	0.009
Disability	1.81 (1.38–2.36)	<0.001	1.65 (1.19–2.28)	0.003	1.44 (1.17–1.78)	0.001	1.43 (1.09–1.88)	0.01
**Health behaviors and health care utilization**								
Obesity	1.16 (0.98–1.36)	0.08	1.15 (0.97–1.37)	0.11	1.16 (1.00–1.35)	0.05	1.09 (0.91–1.32)	0.3
Smoking	1.31 (1.12–1.52)	0.001	1.42 (1.15–1.76)	0.001	1.13 (0.93–1.37)	0.2	1.23 (0.94–1.62)	0.14
Influenza vaccination	1.03 (0.87–1.21)	0.7	1.27 (1.05–1.53)	0.02	1.00 (0.86–1.17)	0.9	1.24 (1.03–1.51)	0.03
Consulted a specialist	1.45 (1.14–1.86)	0.003	1.24 (0.94–1.62)	0.13	1.37 (1.09–1.73)	0.007	1.13 (0.86–1.49)	0.3
Consulted a diabetes specialist	0.98 (0.74–1.30)	0.8	1.01 (0.76–1.33)	0.9	1.16 (0.90–1.49)	0.2	1.38 (1.07–1.78)	0.01
Consulted a cardiologist	1.88 (1.57–2.25)	<0.001	1.86 (1.51–2.29)	<0.001	1.70 (1.44–2.01)	<0.001	2.31 (1.88–2.85)	<0.001
Consulted an ophthalmologist	0.85 (0.72–0.99)	0.049	1.05 (0.86–1.28)	0.6	0.81 (0.69–0.94)	0.007	1.03 (0.85–1.26)	0.7
Performed a cancer screening test	0.84 (0.72–0.98)	0.03	0.96 (0.80–1.15)	0.6	0.86 (0.72–1.01)	0.07	0.88 (0.72–1.06)	0.18
Emergency department visit	1.63 (1.38–1.94)	<0.001	1.44 (1.19–1.73)	<0.001	1.78 (1.52–2.09)	<0.001	1.81 (1.51–2.19)	<0.001

^a^ CI: confidence intervals; CHF: congestive heart failure; IHD: ischemic heart disease; PR: prevalence ratio. Adjusted for the variables in the table.

## Discussion

We examined the correlates of hospitalizations in internal medicine divisions among adults with hypertension, diabetes and CVD, utilizing the database of the largest health maintenance organization in Israel.

Overall utilization of hospitalizations in internal medicine divisions was significantly greater among Arab than Jewish patients, after adjustment for demographic factors, background morbidity and health care utilization patterns. The percentage of patients with at least one hospitalization during a one-year period was higher in Arab than Jewish patients. The higher hospitalization rate among Arab patients was somewhat surprising given their substantially younger age (by 7 years) compared to Jewish patients. Moreover, the percentage of hospitalizations increased significantly already by age 50 years among Arab patients, while among Jewish patients a significant increase was observed from age 70 years. This likely reflects higher NCD burden in Arab patients and greater prevalence of smoking in Arab men; these factors are considered to explain disparities in life expectancy between the Arab and Jewish populations in Israel [10, 14, 15]. Ethnicity was highly correlated with residential SES; therefore, it is difficult to tease-out the specific role of ethnicity vs. SES, especially given the lack of individual level SES indicators in the database. Arab towns in our study clustered in low SES ranks (S2 Fig), with limited variability in this indicator. Among Jewish patients, higher residential SES was inversely related to hospitalizations in internal medicine divisions. Associations of social determinants and health disparities [10–12, 26] with patterns of health service utilization has been described [27–30]. Lower health care utilization in patients residing in low SES communities might be expected in countries without universal health insurance. However, our observation, is surprising, suggesting that in a setting of high access to health care, increased utilization might occur in low SES communities.

In addition to the younger age and lower residential SES, Arab patients displayed a different profile of cardiometabolic diseases. CVD and diabetes were significantly more common among Arab patients while the prevalence of hypertension was similar between the population groups. These observations are in agreement with previous reports by our team and others [10–12].

As expected, high comorbidity burden was positively associated with hospitalizations in both Arab and Jewish patients. Positive associations between multi-morbidity with NCDs and increased risk of hospitalizations and with utilization of other health care services were reported in several countries [31–33]. Obviously, the greater medical needs of patients with multi-morbidity can explain their greater utilization of health care services. Assessing the comorbidity burden is informative; however, from the view of the health care provider and public health, understanding the specific illnesses that contribute to increased hospitalizations is important. In both population groups, we found that several diseases, rather than one specific disease, in addition to having a disability, were associated with increased risk of hospitalizations; the diseases included CVD, kidney disease, mental illnesses and neurodegenerative diseases. These findings are in agreement with previous reports [27, 34, 35], and might explain associations between perceived health status and hospitalization risk [27, 31, 36]. CVD is a major cause of mortality [10]; therefore, a strong relationship between CVD and hospitalizations in internal medicine divisions might be expected. However, CVD comprises various conditions. We found that CHF, arrhythmia, having a history of cardiac catheterization and heart surgery (as indicators of IHD) were significantly positively related to hospitalizations. Possibly, some of the hospitalizations were directly related to treating acute conditions such as worsening of CHF or recurrence of arrhythmia. This is consistent with efforts that are invested in community management of CHF to reduce the burden of recurrent avoidable hospitalizations of these patients. A positive association was found between kidney disease and hospitalizations in internal medicine divisions. Kidney disease is ranked as the 7th leading cause of death in Israel, causing 3.9% of all deaths [37]. Typically, end-stage kidney disease is managed through out-patient nephrology units, with assistance from National Insurance Institute of Israel. Worsening of severe kidney disease can justify hospitalization. Patients with asthma were more often hospitalized than patients without asthma. Niefeld et al. [35] also showed that asthma is a risk factor for preventable hospitalizations in elderly patients with diabetes in the United States [35]. This might be related to less controlled disease [38, 39].

Having a mental illness or a neurodegenerative disease was associated with increased hospitalizations. Increased risk of hospitalization in relation to mental and neurodegenerative illnesses was also shown among elderly diabetic patients [35]. Himelhoch and colleagues [40] showed increased risk of hospitalizations and visits to the emergency room in patients with depression and NCDs compared to patients without these conditions. Depression and other mental illnesses may be related to health care utilization through increasing the severity of background morbidity.

Having a disability was associated with increased hospitalization, in agreement with previous reports [27, 41]. Additionally, smoking was significantly associated with increased hospitalization in both Jewish and Arab men. The prevalence of smoking is highest in Israel among Arab men, estimated at 43.9%, compared to 22.1% and 15.0% in Jewish men and women, respectively; while in Arab women the prevalence of smoking is estimated at 6.7% [10]. Smoking seems to positively correlate with hospitalization, beyond its causative role in NCDs. Our observation is supported by other reports [34, 41].

While associations of sociodemographic factors and NCD comorbidity with the utilization of hospitalizations in internal medicine divisions may be expected, the positive associations observed between utilization of other health care services and hospitalizations, such as previous emergency room visits and consulting with specialist physicians, is of particular interest. These associations could be due to a general tendency towards increased consumption of health services, or simply the tendency of more severe patients to consume health services of different types. Conversely, performing cancer screening tests (i.e., occult stool test, PSA and mammography) and consulting with an ophthalmologist were inversely related to hospitalizations, mainly in the Jewish sample (P = 0.067 and P = 0.009, respectively, for interaction). Likely, performing cancer screening tests and consulting with an ophthalmologist are markers of healthy lifestyle.

Our study has strengths and limitations. We used electronic medical records of patients belonging to the largest HMO in Israel. Differences between physicians in coding diseases may have had an impact on the quality of data, and also differences in documenting disability, smoking and obesity. However, such information bias is likely non-differential, thus leading the association measure towards the null. Individual-level information on SES was lacking. The sample size was large, thus enabling us to conduct this comprehensive assessment of correlates of hospitalizations, and to identify important associations and interactions. We focused on one region in Israel. This was stimulated by known regional variation in health indicators and socioeconomic factors. Our findings may be generalizable to other populations with similar characteristics and health care systems.

In conclusion, in a country with universal health insurance, associations were found of sociodemographic factors, morbidity, health behaviors and health service use with hospitalization in internal medicine divisions. Socioeconomic disparities might account for ethnic differences in hospitalization patterns. Several NCDs, rather than one specific disease, in addition to disability and smoking, were related to hospitalization use; these factors should be targeted in an effort to reduce healthcare costs related to hospitalizations.

## Supporting information

S1 Checklist STROBE checklist. (DOCX)

S1 Table List of variables analyzed in the study. CVD: cardiovascular disease; IHD: ischemic heart disease; SES: socioeconomic status.
(DOCX)

S2 Table Definition of composite variables. CVD: cardiovascular disease: PSA: prostatic specific antigen.
(DOCX)

S3 Table Multivariable analysis of the correlates of hospitalizations in internal medicine divisions among patients aged ≥40 years with cardiovascular disease, diabetes or hypertension. CI: confidence intervals; CHF; congestive heart failure; IHD: ischemic heart disease; PR: prevalence ratio; SES: socioeconomic status. Adjusted for the variables in the table. This pooled analysis of both population groups (Arab and Jewish patients) that included the variable residential SES instead of population group.
(DOCX)

S4 Table Sensitivity analyses of pooled multivariable models of the correlates of hospitalizations in internal medicine divisions with interaction terms. CI: confidence intervals; CHF; congestive heart failure; IHD: ischemic heart disease; PR: prevalence ratio. Adjusted for the variables in the table.
(DOCX)

S1 Fig Correlation matrix of the independent variables. Data presented are correlation coefficients: green gradient represents positive correlation, red gradient represents negative correlation, yellow ~0 (no correlation) CVD: cardiovascular disease; CHF: congestive heart failure; DM: diabetes mellitus; ER: emergency room department; IHD: ischemic heart disease; SES: socioeconomic status.
(TIF)

S2 Fig Residential socioeconomic status of Jewish participants (A) and Arab participants (B).
(TIF)
