# Supplementary material for: Correlates of hospitalizations in internal medicine divisions among Israeli adults of different ethnic groups with hypertension, diabetes and cardiovascular diseases
Source: PLoS One. 2019 Apr 24;14(4):e0215639. doi: 10.1371/journal.pone.0215639 (PMC6481835; doi:10.1371/journal.pone.0215639)
Supplement: S1 Checklist — (DOCX) [file pone.0215639.s001.docx]

STROBE Statement—checklist of items that should be included in reports of observational studies

|  | Item No. | Recommendation | Page  No. | Relevant text from manuscript |
| --- | --- | --- | --- | --- |
| **Title and abstract** | 1 | (*a*) Indicate the study’s design with a commonly used term in the title or the abstract | 2 | A cross-sectional study was conducted |
|  |  | (*b*) Provide in the abstract an informative and balanced summary of what was done and what was found | 2 |  |
| Introduction | | | |  |
| Background/rationale | 2 | Explain the scientific background and rationale for the investigation being reported | 4-5 |  |
| Objectives | 3 | State specific objectives, including any prespecified hypotheses | 5 | The aim of the current study was to compare the correlates of hospitalizations in internal medicine divisions between Arab and Jewish patients with chronic diseases: CVD, diabetes and hypertension, in Israel. Our hypothesis was that demographic, clinical and behavioral characteristics (e.g. comorbidities, health care utilization patterns) are related to hospitalizations, and that these might differ according to ethnic group. |
| Methods | | | |  |
| Study design | 4 | Present key elements of study design early in the paper | 5 | A cross-sectional study was conducted…. |
| Setting | 5 | Describe the setting, locations, and relevant dates, including periods of recruitment, exposure, follow-up, and data collection | 5-6 | adults aged 40 years or above from the Hadera sub-district, utilizing the database of the Sharon-Shomron sub-district of Clalit Health Services [[23](#_ENREF_23)]. Clalit is the largest health maintenance organization (HMO) in Israel, insuring about 4.2 million members; i.e. ~52% of the Israeli population…. |
| Participants | 6 | *Cross-sectional study*—Give the eligibility criteria, and the sources and methods of selection of participants | 5 | Study eligibility criteria included: CVD, hypertension or diabetes, as documented in the Clalit database by primary care physicians’ reports at any time before or during 2008 (S1 and S2 Tables) [[24](#_ENREF_24)]. This yielded a total of 31,883 persons. We excluded from the study 3471 patients with a diagnosis of cancer and 19 patients without information on ethnicity. |
| Variables | 7 | Clearly define all outcomes, exposures, predictors, potential confounders, and effect modifiers. Give diagnostic criteria, if applicable | 6 | Definitions of the dependent variable…  Independent variables… |
| Data sources/ measurement | 8* | For each variable of interest, give sources of data and details of methods of assessment (measurement). Describe comparability of assessment methods if there is more than one group | *6 & S1Table* |  |
| Bias | 9 | Describe any efforts to address potential sources of bias | 6 | Documentation of hospitalizations is based on payments of the HMO to the hospitals for these services, therefore the accuracy is high. |
| Study size | 10 | Explain how the study size was arrived at | *5* | Data for all subjects meeting the inclusion criteria were used |
| Continued on next page Quantitative variables | 11 | Explain how quantitative variables were handled in the analyses. If applicable, describe which groupings were chosen and why | *6 & S1Table* | Age was grouped as 40-44, 45-49, 50-54, 55-59, 60-64, 65-69, 70-74 and ≥75). |
| Statistical methods | 12 | (*a*) Describe all statistical methods, including those used to control for confounding | 7 |  |
|  |  | (*b*) Describe any methods used to examine subgroups and interactions | 7 |  |
|  |  | (*c*) Explain how missing data were addressed |  |  |
|  |  | *Cross-sectional study*—If applicable, describe analytical methods taking account of sampling strategy | Not applicable |  |
|  |  | (*e*) Describe any sensitivity analyses | 7 |  |
| Results | | | | |
| Participants | 13* | (a) Report numbers of individuals at each stage of study—eg numbers potentially eligible, examined for eligibility, confirmed eligible, included in the study, completing follow-up, and analysed | 7 |  |
|  |  | (b) Give reasons for non-participation at each stage | 5 |  |
|  |  | (c) Consider use of a flow diagram |  |  |
| Descriptive data | 14* | (a) Give characteristics of study participants (eg demographic, clinical, social) and information on exposures and potential confounders | 8-9 |  |
|  |  | (b) Indicate number of participants with missing data for each variable of interest |  |  |
| Outcome data | 15* | *Cross-sectional study—*Report numbers of outcome events or summary measures | *Page 8 Table 1* |  |
| Main results | 16 | (*a*) Give unadjusted estimates and, if applicable, confounder-adjusted estimates and their precision (eg, 95% confidence interval). Make clear which confounders were adjusted for and why they were included | Table1-5 and relevant text |  |
|  |  | (*b*) Report category boundaries when continuous variables were categorized | Table 1 |  |
|  |  | (*c*) If relevant, consider translating estimates of relative risk into absolute risk for a meaningful time period |  |  |

| Other analyses | 17 | Report other analyses done—eg analyses of subgroups and interactions, and sensitivity analyses | S1 fig  S2 fig  Table 4  S4 Table |  |
| --- | --- | --- | --- | --- |
| Discussion | | | | |
| Key results | 18 | Summarise key results with reference to study objectives | Page 19 |  |
| Limitations | 19 | Discuss limitations of the study, taking into account sources of potential bias or imprecision. Discuss both direction and magnitude of any potential bias | Page 22 | Our study has strengths and limitations. |
| Interpretation | 20 | Give a cautious overall interpretation of results considering objectives, limitations, multiplicity of analyses, results from similar studies, and other relevant evidence | Page 22 |  |
| Generalisability | 21 | Discuss the generalisability (external validity) of the study results | Page 22 | We believe that our findings may be generalizable to other populations with similar characteristics and health care system. |
| Other information | |  | | |
| Funding | 22 | Give the source of funding and the role of the funders for the present study and, if applicable, for the original study on which the present article is based | Page 24 | This study was funded by the Israel National Institute for Health Policy and Health Services Research awarded to Dr. K. Muhsen (Award number 32/2007A). The funders were not involved in the study design, data collection and analysis, the decision to publish, or the preparation of the manuscript. |

*Give information separately for cases and controls in case-control studies and, if applicable, for exposed and unexposed groups in cohort and cross-sectional studies.

**Note:** An Explanation and Elaboration article discusses each checklist item and gives methodological background and published examples of transparent reporting. The STROBE checklist is best used in conjunction with this article (freely available on the Web sites of PLoS Medicine at http://www.plosmedicine.org/, Annals of Internal Medicine at http://www.annals.org/, and Epidemiology at http://www.epidem.com/). Information on the STROBE Initiative is available at www.strobe-statement.org.
