# Supplementary material for: Correlates of hospitalizations in internal medicine divisions among Israeli adults of different ethnic groups with hypertension, diabetes and cardiovascular diseases
Source: PLoS One. 2019 Apr 24;14(4):e0215639. doi: 10.1371/journal.pone.0215639 (PMC6481835; doi:10.1371/journal.pone.0215639)
Supplement: S1 Table — CVD: cardiovascular disease; IHD: ischemic heart disease; SES: socioeconomic status. (DOCX) [file pone.0215639.s002.docx]

**S1 Table: List of variables analyzed in the study**

| Variables | Variable type |
| --- | --- |
| Socio-demographic variables |  |
| Sex | Dichotomous, males, females |
| Population group | Dichotomous, Arab or Jews |
| Age, years | Continuous and categorical (5-years grouping) |
| Residential SES | Discrete, scale 1-10 |
| Background morbidity |  |
| Hypertension | Dichotomous, yes=1, n=0 |
| Diabetes | Dichotomous, yes=1, n=0 |
| CVD | Dichotomous, yes=1, n=0 |
| IHD | Dichotomous, yes=1, n=0 |
| CHF | Dichotomous, yes=1, n=0 |
| Arrhythmia | Dichotomous, yes=1, n=0 |
| Cardiomyopathy | Dichotomous, yes=1, n=0 |
| Carotid artery disease | Dichotomous, yes=1, n=0 |
| Mental illness | Composite variable, dichotomous**,**  yes=1, n=0 |
| Kidney disease | Composite variable, dichotomous**,**  yes=1, n=0 |
| Neurodegenerative disease | Composite variable, dichotomous**,**  yes=1, n=0 |
| Hyperlipidemia | Dichotomous, yes=1, n=0 |
| Asthma | Dichotomous, yes=1, n=0 |
| Stroke | Dichotomous, yes=1, n=0 |
| Disability | Dichotomous, yes=1, n=0 |
| Cardiac catheterization | Dichotomous, yes=1, n=0 |
| Past heart surgery | Dichotomous, yes=1, n=0 |
| Comorbidity score | Composite variable, discrete |
| Health behavior and health care utilization patterns |  |
| Obesity | Dichotomous, yes=1, n=0 |
| Smoking | Dichotomous, yes=1, n=0 |
| Disability | Dichotomous, yes=1, n=0 |
| Received influenza vaccine | Dichotomous, yes=1, n=0 |
| Performed HbA1c test | Dichotomous, yes=1, n=0 |
| Consulted a specialist | Composite variable, dichotomous, yes=1, n=0 |
| Consulted a diabetes specialist | Dichotomous, yes=1, n=0 |
| Consulted a cardiologist | Dichotomous, yes=1, n=0 |
| Consult an ophthalmologist | Dichotomous, yes=1, n=0 |
| Consulted a surgeon | Dichotomous, yes=1, n=0 |
| Performed a cancer screening test, 2008 | Composite variable, dichotomous |
| Emergency department visit, 2008 | Dichotomous, yes=1, n=0 |

This is the S1 Table legend:

CHF: congestive heart failure; CVD: cardiovascular disease; IHD: ischemic heart disease; SES: Socioeconomic status
