# Supplementary material for: Correlates of hospitalizations in internal medicine divisions among Israeli adults of different ethnic groups with hypertension, diabetes and cardiovascular diseases
Source: PLoS One. 2019 Apr 24;14(4):e0215639. doi: 10.1371/journal.pone.0215639 (PMC6481835; doi:10.1371/journal.pone.0215639)
Supplement: S2 Table — CVD: cardiovascular disease: PSA: prostatic specific antigen. (DOCX) [file pone.0215639.s003.docx]

**S2 Table: Definition of composite variables**

| Composite variables |
| --- |
| Cardiovascular disease (CVD): having any of the following diagnoses:  ischemic heart disease/ congestive heart failure/ arrhythmia/ pulmonary hypertension/ cardiomyopathy/ carotid artery disease/ stroke |
| Mental illness: having any of the following diagnoses:  anxiety/ neuroses/ depression/ bipolar disease/ psychoses |
| Kidney disease: having any of the following diagnoses:  dialysis/ chronic renal failure/ kidney transplant |
| Neurodegenerative disease: having any of the following diagnoses: dementia/Alzheimer's disease/ Parkinson’s disease |
| Comorbidity score: summative score:  One point was given for each of the following diseases: CVD/asthma/hyperlipidemia/mental illness/neurodegenerative disease, and two points for kidney disease |
| Consulted a specialist: consulted with any of the following specialists during 2008  Consulted a diabetes specialist; consulted a cardiologist; consulted an ophthalmologist; consulted a surgeon; consulted an orthopedist |
| Performed any cancer screening test: performed any of the following during 2008  Performed an occult blood test, mammography (women), PSA (men) |

This is the S2 Table legend: CVD: cardiovascular disease: PSA: prostatic specific antigen
