# Supplementary material for: Correlates of hospitalizations in internal medicine divisions among Israeli adults of different ethnic groups with hypertension, diabetes and cardiovascular diseases
Source: PLoS One. 2019 Apr 24;14(4):e0215639. doi: 10.1371/journal.pone.0215639 (PMC6481835; doi:10.1371/journal.pone.0215639)
Supplement: S3 Table — CI: confidence intervals; CHF; congestive heart failure; IHD: ischemic heart disease; PR: prevalence ratio; SES: socioeconomic status. Adjusted for the variables in the table. This pooled analysis of both population groups (Arab and Jewish patients) that included the variable residential SES instead of population group. (DOCX) [file pone.0215639.s004.docx]

**S3 Table: Multivariable analysis of the correlates of hospitalizations in internal medicine divisions among patients aged ≥40 years with cardiovascular disease, diabetes or hypertension**

|  | **Adjusted PR** **(95% CI)** | **P** |
| --- | --- | --- |
| **Sociodemographic variables** |  |  |
| SES place of residence (discrete variable) | 0.94 (0.92-0.97) | <0.001 |
| Sex (women vs. men) | 1.06 (0.97-1.17) | 0.22 |
| Age in years (a continuous variable) | 1.01 (1.01-1.02) | <0.001 |
| **Background morbidity** |  |  |
| Diabetes | 1.09 (0.99-1.18) | 0.06 |
| CHF | 1.49 (1.31-1.69) | <0.001 |
| Arrhythmia | 1.56 (1.41-1.73) | <0.001 |
| IHD | 1.14 (1.04-1.26) | 0.007 |
| Kidney disease | 1.50 (1.32-1.71) | <0.001 |
| Asthma | 1.33 (1.17-1.50) | <0.001 |
| Stroke | 1.63 (1.47-1.81) | <0.001 |
| Neurodegenerative disease | 1.44 (1.23-1.70) | <0.001 |
| Heart surgery | 1.49 (1.10-2.01) | 0.009 |
| Cardiac catheterization | 1.74 (1.55-1.96) | <0.001 |
| Mental illness | 1.35 (1.22-1.49) | <0.001 |
| Disability | 1.60 (1.41-1.81) | <0.001 |
| **Health behaviors and health care utilization** |  |  |
| Obesity | 1.14 (1.05-1.24) | 0.002 |
| Smoking | 1.28 (1.17-1.41) | <0.001 |
| Consulted a cardiologist | 1.89 (1.72-2.07) | <0.001 |
| Consulted a diabetes specialist | 1.15 (1.01-1.31) | 0.04 |
| Consulted an ophthalmologist | 0.90 (0.82-0.98) | 0.01 |
| Consulted a specialist | 1.30 (1.15-1.47) | <0.001 |
| Influenza vaccination | 1.11 (1.02-1.21) | 0.02 |
| Performed any screening test | 0.86 (0.79-0.93) | <0.001 |
| Emergency department visit | 1.67 (1.53-1.82) | <0.001 |

### This is the S3 Table legend

CI: confidence intervals; CHF; congestive heart failure; IHD: ischemic heart disease; PR: prevalence ratio; SES: socioeconomic status

Adjusted for the variables in the table.

This pooled analysis of both population groups (Arab and Jewish patients) that included the variable residential SES instead of population group
