# Supplementary material for: Correlates of hospitalizations in internal medicine divisions among Israeli adults of different ethnic groups with hypertension, diabetes and cardiovascular diseases
Source: PLoS One. 2019 Apr 24;14(4):e0215639. doi: 10.1371/journal.pone.0215639 (PMC6481835; doi:10.1371/journal.pone.0215639)
Supplement: S4 Table — CI: confidence intervals; CHF; congestive heart failure; IHD: ischemic heart disease; PR: prevalence ratio. Adjusted for the variables in the table. (DOCX) [file pone.0215639.s005.docx]

**S4 Table: Sensitivity analyses of pooled multivariable models of the correlates of hospitalizations in internal medicine divisions with interaction terms**

|  | **Model 1** |  | **Model 2** |  | **Model 3** |  | **Model 4** |  | **Model 5** |  | **Model 6** |  |
| --- | --- | --- | --- | --- | --- | --- | --- | --- | --- | --- | --- | --- |
|  | **Adjusted PR** **(95% CI)** | **P** | **Adjusted PR** **(95% CI)** | **P** | **Adjusted PR** **(95% CI)** | **P** | **Adjusted PR** **(95% CI)** | **P** | **Adjusted PR** **(95% CI)** | **P** | **Adjusted PR** **(95% CI)** | **P** |
| **Sociodemographic variables** |  |  |  |  |  |  |  |  |  |  |  |  |
| Population group, Arab vs. Jewish patients | 1.13 (1.02-1.26) | 0.025 | 1.17 (1.06-1.28) | 0.001 | 1.08 (0.95-1.22) | 0.2 | 1.13 (1.02-1.26) | 0.019 | 1.13 (1.02-1.27) | 0.02 | 1.17 (1.05-1.30) | 0.004 |
| Sex (women vs. men) | 1.06 (0.97-1.16) | 0.2 | 1.06 (0.97-1.16) | 0.19 | 1.06 (0.97-1.16) | 0.18 | 1.06 (0.97-1.16) | 0.20 | 1.06 (0.97-1.16) | 0.18 | 1.05 (0.96-1.15) | 0.2 |
| Age in years (a continuous variable) | 1.01 (1.01-1.02) | <0.001 | 1.01 (1.01-1.02) | <0.001 | 1.01 (1.01-1.02) | <0.001 | 1.01 (1.01-1.02) | <0.001 | 1.01 (1.01-1.02) | <0.001 | 1.01 (1.01-1.02) | <0.001 |
| **Background morbidity** |  |  |  |  |  |  |  |  |  |  |  |  |
| Diabetes | 1.09 (1.00-1.18) | 0.046 | 1.09 (1.00-1.18) | 0.043 | 1.08 (0.99-1.20) | 0.068 | 1.09 (1.00-1.20) | 0.050 | 1.08 (0.99-1.17) | 0.071 | 1.08 (0.99-1.17) | 0.058 |
| IHD | 1.02 (0.91-1.14) | 0.7 | 1.10 (1.00-1.21) | 0.046 | 1.10 (0.99-1.20) | 0.056 | 1.10 (1.00-1.21) | 0.044 | 1.10 (1.00-1.21) | 0.045 | 1.10 (1.00-1.21) | 0.049 |
| CHF | 1.51 (1.33-1.70) | <0.001 | 1.52 (1.34-1.71) | <0.001 | 1.52 (1.34-1.71) | <0.001 | 1.51 (1.33-1.70) | <0.001 | 1.51 (1.34-1.71) | <0.001 | 1.52 (1.35-1.72) | <0.001 |
| Arrhythmia | 1.60 (1.46-1.77) | <0.001 | 1.61 (1.46-1.77) | <0.001 | 1.60 (1.46-1.77) | <0.001 | 1.61 (1.46-1.77) | <0.001 | 1.60 (1.45-1.77) | <0.001 | 1.60 (1.45-1.76) | <0.001 |
| Stroke | 1.61 (1.45-1.78) | <0.001 | 1.62 (1.46-1.79) | <0.001 | 1.60 (1.45-1.77) | <0.001 | 1.61 (1.46-1.78) | <0.001 | 1.61 (1.46-1.78) | <0.001 | 1.61 (1.45-1.78) | <0.001 |
| Cardiac catheterization | 1.75 (1.56-1.96) | <0.001 | 1.53 (1.33-1.77) | <0.001 | 1.76 (1.57-1.97) | <0.001 | 1.75 (1.56-1.96) | <0.001 | 1.75 (1.57-1.96) | <0.001 | 1.75 (1.57-1.96) | <0.001 |
| Past heart surgery | 1.52 (1.14-2.04) | 0.004 | 1.53 (1.14-2.04) | 0.004 | 1.52 (1.13-2.02) | 0.005 | 1.52 (1.14-2.03) | 0.005 | 1.52 (1.14-2.03) | 0.004 | 1.52 (1.14-2.03) | 0.005 |
| Kidney disease | 1.46 (1.29-1.65) | <0.001 | 1.46 (1.29-1.65) | <0.001 | 1.45 (1.29-1.64) | <0.001 | 1.46 (1.29-1.65) | <0.001 | 1.45 (1.29-1.64) | <0.001 | 1.46 (1.29-1.65) | <0.001 |
| Neurodegenerative disease | 1.44 (1.23-1.68) | <0.001 | 1.43 (1.22-1.67) | <0.001 | 1.45 (1.24-1.69) | <0.001 | 1.43 (1.22-1.67) | <0.001 | 1.43 (1.22-1.67) | <0.001 | 1.43 (1.23-1.67) | <0.001 |
| Asthma | 1.27 (1.13-1.43) | <0.001 | 1.27 (1.13-1.44) | <0.001 | 1.27 (1.13-1.43) | <0.001 | 1.27 (1.13-1.44) | <0.001 | 1.28 (1.13-1.44) | <0.001 | 1.28 (1.13-1.44) | <0.001 |
| Mental illness | 1.36 (1.23-1.50) | <0.001 | 1.36 (1.24-1.50) | <0.001 | 1.36 (1.24-1.50) | <0.001 | 1.36 (1.24-1.50) | <0.001 | 1.37 (1.24-1.50) | <0.001 | 1.36 (1.23-1.50) | <0.001 |
| Disability | 1.61 (1.42-1.82) | <0.001 | 1.62 (1.43-1.83) | <0.001 | 1.60 (1.42-1.81) | <0.001 | 1.62 (1.43-1.83) | <0.001 | 1.61 (1.43-1.82) | <0.001 | 1.62 (1.43-1.83) | <0.001 |
| **Health behaviors and health care utilization** |  |  |  |  |  |  |  |  |  |  |  |  |
| Obesity | 1.16 (1.07-1.25) | <0.001 | 1.16 (1.07-1.25) | <0.001 | 1.15 (1.06-1.25) | <0.001 | 1.16 (1.07-1.25) | <0.001 | 1.15 (1.07-1.25) | <0.001 | 1.15 (1.07-1.25) | <0.001 |
| Smoking | 1.29 (1.18-1.40) | <0.001 | 1.29 (1.18-1.40) | <0.001 | 1.29 (1.18-1.41) | <0.001 | 1.29 (1.18-1.41) | <0.001 | 1.29 (1.18-1.42) | <0.001 | 1.30 (1.19-1.42) | <0.001 |
| Influenza vaccination | 1.10 (1.01-1.19) | 0.021 | 1.08 (0.95-1.22) | 0.2 | 1.00 (0.90-1.11) | 0.9 | 1.10 (1.02-1.20) | 0.019 | 1.11 (1.02-1.20) | 0.014 | 1.11 (1.02-1.20) | 0.016 |
| Consulted a specialist | 1.30 (1.15-1.47) | 0.003 | 1.30 (1.15-1.47) | 0.003 | 1.30 (1.15-1.47) | 0.001 | 1.30 (1.15-1.46) | <0.001 | 1.29 (1.15-1.46) | <0.001 | 1.30 (1.16-1.47) | <0.001 |
| Consulted a diabetes specialist | 1.15 (1.01-1.30) | 0.029 | 1.15 (1.01-1.30) | 0.035 | 1.15 (1.02-1.31) | 0.027 | 1.15 (1.01-1.30) | 0.033 | 1.15 (1.01-1.30) | 0.033 | 1.16 (1.02-1.31) | 0.025 |
| Consulted a cardiologist | 1.92 (1.75-2.10) | <0.001 | 1.92 (1.75-2.10) | <0.001 | 1.91 (1.75-2.10) | <0.001 | 1.76 (1.58-1.96) | <0.001 | 1.92 (1.75-2.10) | <0.001 | 1.91 (1.75-2.10) | <0.001 |
| Consulted an ophthalmologist | 0.89 (0.82-0.97) | <0.001 | 0.89 (0.82-0.97) | 0.008 | 0.89 (0.82-0.97) | 0.009 | 0.89 (0.82-0.97) | 0.01 | 0.83 (0.75-0.91) | <0.001 | 0.89 (0.82-0.97) | <0.001 |
| Performed a cancer screening test | 0.85 (0.79-0.93) | <0.001 | 0.85 (0.79-0.93) | <0.001 | 0.85 (0.79-0.92) | <0.001 | 0.86 (0.79-0.93) | <0.001 | 0.85 (0.79-0.93) | <0.001 | 0.81 (0.73-0.89) | <0.001 |
| Emergency department visit | 1.67 (1.53-1.81) | <0.001 | 1.67 (1.54-1.82) | <0.001 | 1.67 (1.54-1.82) | <0.001 | 1.67 (1.54-1.82) | <0.001 | 1.67 (1.54-1.82) | <0.001 | 1.67 (1.54-1.82) | <0.001 |
| **Interaction terms** |  |  |  |  |  |  |  |  |  |  |  |  |
| Population group 1= Arabs ˟ IHD | 1.23 (1.05-1.43) | 0.009 |  |  |  |  |  |  |  |  |  |  |
| Population group 1= Arabs˟ Cardiac catheterization |  |  | 1.36 (1.11-1.64) | 0.002 |  |  |  |  |  |  |  |  |
| Population group 1= Arabs˟ Influenza vaccination |  |  |  |  | 1.28 (1.09-1.50) | 0.002 |  |  |  |  |  |  |
| Population group 1= Arabs˟ consulted a cardiologist |  |  |  |  |  |  | 1.24 (1.06-1.45) | 0.007 |  |  |  |  |
| Population group 1= Arabs˟ consulted an ophthalmologist |  |  |  |  |  |  |  |  | 1.23 (1.05-1.43) | 0.009 |  |  |
| Population group 1= Arabs˟ Performed a cancer screening test |  |  |  |  |  |  |  |  |  |  | 1.16 (0.99-1.36) | 0.067 |

This is S4 Table legend

CI: confidence intervals; CHF; congestive heart failure; IHD: ischemic heart disease; PR: prevalence ratio. Adjusted for the variables in the table.
