# Supplementary figures and images for: Correlates of hospitalizations in internal medicine divisions among Israeli adults of different ethnic groups with hypertension, diabetes and cardiovascular diseases
Source: PLoS One. 2019 Apr 24;14(4):e0215639. doi: 10.1371/journal.pone.0215639 (PMC6481835; doi:10.1371/journal.pone.0215639)

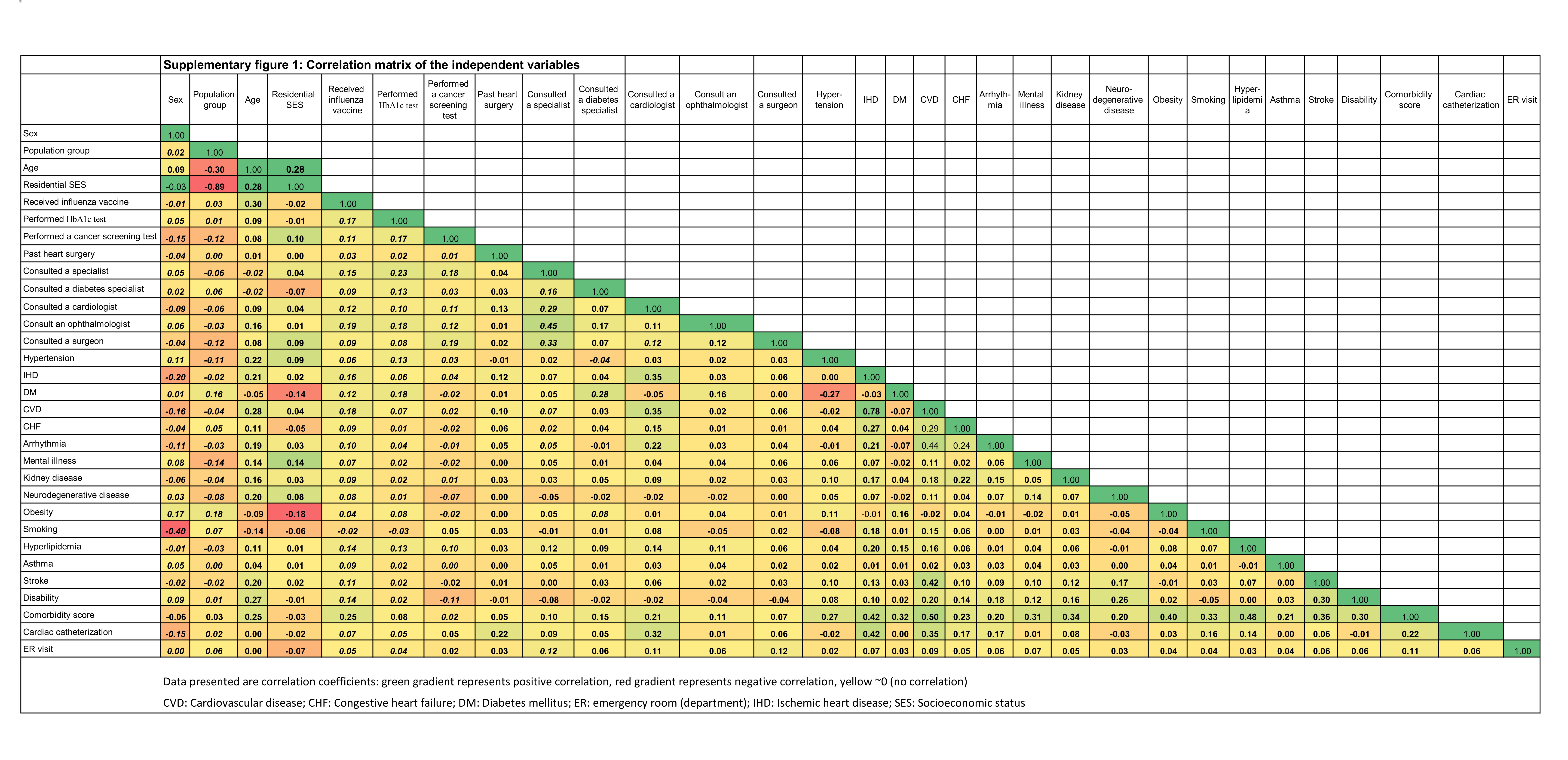

Supplement: S1 Fig — Data presented are correlation coefficients: green gradient represents positive correlation, red gradient represents negative correlation, yellow ~0 (no correlation) CVD: cardiovascular disease; CHF: congestive heart failure; DM: diabetes mellitus; ER: emergency room department; IHD: ischemic heart disease; SES: socioeconomic status. (TIF) [file pone.0215639.s006.tif]

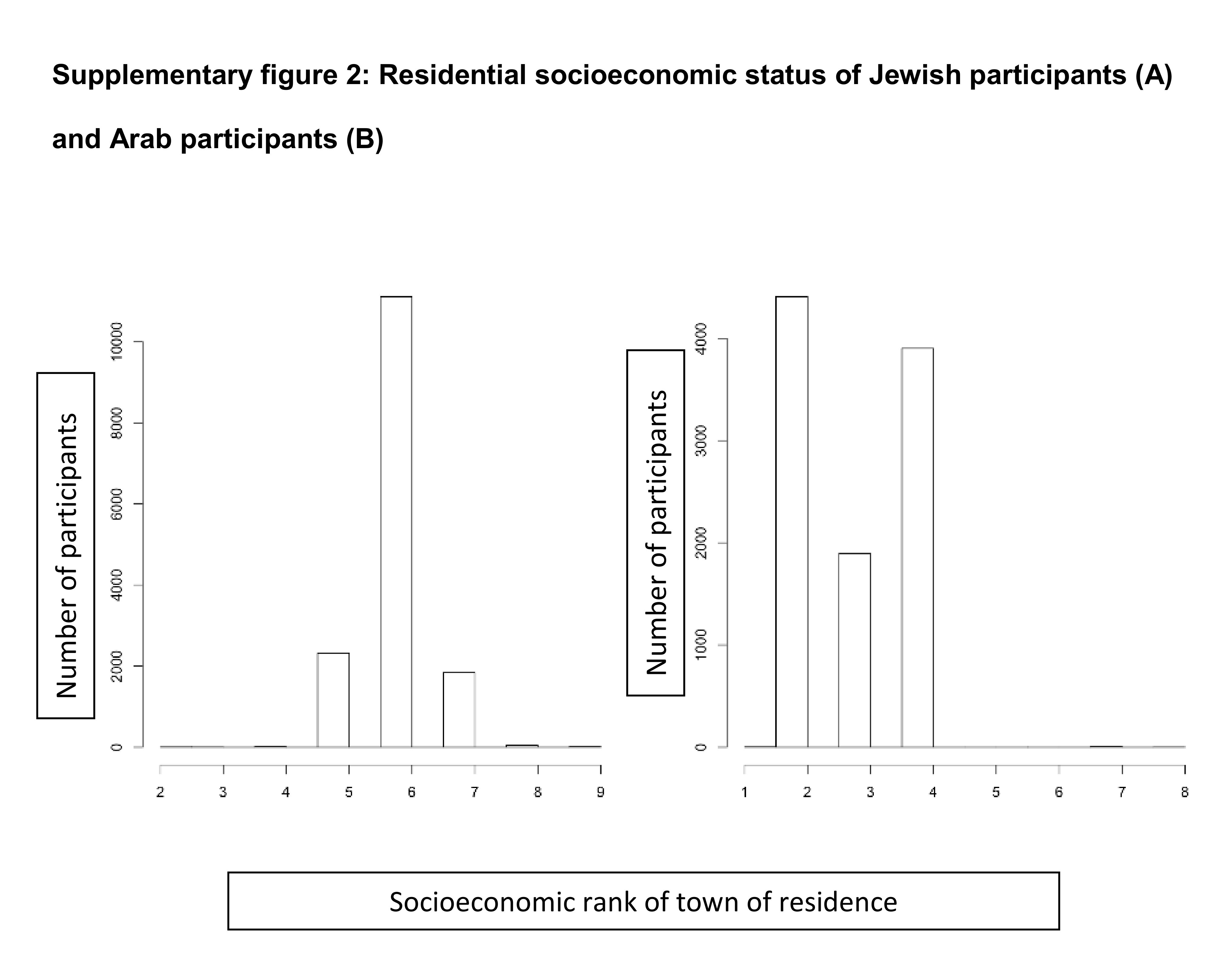

Supplement: S2 Fig — Residential socioeconomic status of Jewish participants (A) and Arab participants (B). (TIF) [file pone.0215639.s007.tif]
